# At-Line Monitoring of the Extraction Process of Rosmarini Folium via Wet Chemical Assays, UHPLC Analysis, and Newly Developed Near-Infrared Spectroscopic Analysis Methods

**DOI:** 10.3390/molecules24132480

**Published:** 2019-07-06

**Authors:** Stefanie Delueg, Christian G. Kirchler, Florian Meischl, Yukihiro Ozaki, Michael A. Popp, Günther K. Bonn, Christian W. Huck

**Affiliations:** 1 Institute of Analytical Chemistry and Radiochemistry, University of Innsbruck, Innrain 80/82, 6020 Innsbruck, Austria; 2 School of Science and Technology, Kwansei Gakuin University, Gakuen, Sanda, Hyogo 669-1337, Japan; 3 Michael Popp Research Institute for New Phyto Entities, University of Innsbruck, Mitterweg 24, 6020 Innsbruck, Austria; 4 ADSI–Austrian Drug Screening Institute GmbH, Innrain 66a, 6020 Innsbruck, Austria

**Keywords:** ultra-high performance liquid chromatography, Folin–Ciocalteu, total hydroxycinnamic derivatives, phytoextraction, near-infrared spectroscopy

## Abstract

The present study demonstrates the applicability of at-line monitoring of the extraction process of *Rosmarinus officinalis* L. leaves (Rosmarini folium) and the development of near-infrared (NIR) spectroscopic analysis methods. Therefore, whole dried Rosmarini folium samples were extracted by maceration with 70% (*v*/*v*) ethanol. For the experimental design three different specimen-taking plans were chosen. At first, monitoring was carried out using three common analytical methods: (a) total hydroxycinnamic derivatives according to the European Pharmacopoeia, (b) total phenolic content according to Folin–Ciocalteu, and (c) rosmarinic acid content measured by UHPLC-UV analysis. Precision validation of the wet chemical assays revealed a repeatability of (a) 0.12% relative standard deviation (RSD), (b) 1.1% RSD, and (c) 0.28% RSD, as well as an intermediate precision of (a) 4.1% RSD, (b) 1.3% RSD, and (c) 0.55% RSD. The collected extracts were analyzed with a NIR spectrometer using a temperature-controlled liquid attachment. Samples were measured in transmission mode with an optical path length of 1 mm. The combination of the recorded spectra and the previously obtained analytical reference values in conjunction with multivariate data analysis enabled the successful establishment of partial least squares regression (PLSR) models. Coefficients of determination (R^2^) were: (a) 0.94, (b) 0.96, and (c) 0.93 (obtained by test-set validation). Since Pearson correlation analysis revealed that the reference analyses correlated with each other just one of the PSLR models is required. Therefore, it is suggested that PLSR model (b) be used for monitoring the extraction process of Rosmarini folium. The application of NIR spectroscopy provides a fast and non-invasive alternative analysis method, which can subsequently be implemented for on- or in-line process control.

## 1. Introduction

Plants have been the main source of traditional medicine systems over millennia and are still of great importance in healthcare today [1,2]. The demand for pharmaceuticals based on natural sources has even increased in recent times [3,4]. In Europe, herbal substances, preparations, and combinations are assessed and regulated by the Committee on Herbal Medicinal Products (HMPC), which is part of the European Medicines Agency (EMA), and the European Pharmacopoeia (Ph. Eur.) [5,6]. Nevertheless, chemically complex plant-based preparations are in constant competition with chemically defined products. Therefore, quality assurance and analytics of these so-called “phytopharmaceuticals” is a big challenge for the manufacturers. Besides the incoming goods, inspection and extraction control of medicinal plants play an important role in the yield and purity of the product [7]. Furthermore, resource and cost efficiency can be increased by extraction optimization. Near-infrared (NIR) spectroscopy and Raman spectroscopy represent attractive analysis techniques for the research demand regarding the at-line, on-line, or in-line analysis of phytoextraction processes [3,8,9,10,11,12,13,14]. In contrast to common off-line reference analyses, NIR spectroscopic process monitoring as process analytical technology (PAT) has convincing advantages since its operation is non-destructive, contact-free, pollution-free, does not require any additional solvents, saves energy, and is highly cost-effective. The recorded NIR spectra include multiple physical and chemical parameters which can be determined simultaneously. The use of optical light fibers facilitates a distance of up to several hundred meters between the measurement probe and the analyzer. Furthermore, NIR spectroscopy fulfills the requirements of fast real-time process control. Nevertheless, the development of a NIR spectroscopic analysis method is time- and resource-consuming and has to be undertaken by experienced professionals [15]. As for reference analytics, the quantification of the total phenolic compound is specified by the European Pharmacopoeia. The antioxidant properties of certain phytogenic substances are attributed to the presence of phenol terpens in rosemary [16]. The analysis described in the European Pharmacopoeia is principally for the analysis of cinnamic acid derivatives. The assay is complicated and another wet chemical assay (Folin–Ciocalteu) has to be executed to verify the results. The Folin–Ciocalteu analysis is not that specific but is more reproducible. However, HPLC analysis is currently the method of choice. It is state-of-the-art, since the analyses can be measured without any major work-up and the measurement can be automated, in contrast to the wet-chemical investigations [17]. In order to meet the requirements of the EMA and still be up to date, all three analyses were carried out, calibrated into the system, and checked for reproducibility, traceability and comparability. Thus, a holistic view of the system and the determination of the saturation of the extraction could be determined. The present feasibility study reports the monitoring of the phytoextraction process of *Rosmarinus officinalis* L. leaves using common analytical methods as well as newly developed NIR spectroscopic methods applying partial least squares regression (PLSR) models as multivariate data analysis (MVA) tools. This analysis was used as the basis for an online fixation of NIR measurements in phytochemical extractions.

## 2. Results and Discussion

### 2.1. Wet Chemical Assays (European Pharmacopoeia and Folin–Ciocalteu)

The wet chemical assays for the determination of total hydroxycinnamic derivatives (THCD) according to Ph. Eur. and gallic acid equivalents (GAE%) referred to as Folin–Ciocalteu (FC) have similar reaction mechanisms. The chemical background is very complex and not yet fully understood. Both wet chemical assays are based on the reduction of a mixture composed of tungsten and molybdenum oxides [18]. In the fully oxidized valence state the isopolyphosphotungstates are colorless and the molybdenum compounds are yellow. The reagent mixture consists of heteropolyphosphotungstates-molybdates. In an acidic solution a hydrated octahedral complex of metal oxides, which is coordinated around a central phosphate, appears. Due to the reversible reduction of one or two electrons the color of the solution changes. In the case of the Ph. Eur. assay the solution turns red and in the case of the FC assay it turns blue [19]. The more intense the color the higher the concentration of the phenolic compounds is in the samples.

The Ph. Eur. assay, which can be assigned to the THCD, is more substance-specific than the FC assay. This is based on the different chemicals which are added for the assays. FC targets hydroxy groups, whereas the Ph. Eur. assay targets carboxyl groups which are not as common as hydroxy groups in the chemistry of natural products [20]. In the present study both assays were applied for monitoring the extraction process of Rosmarini folium. Correlation analyses of the two assays revealed a Pearson correlation of 0.966 (Table 1). This means that substances which were assessed by measurement via the Ph. Eur. assay were highly correlated with those measured by the FC assay, and vice versa.

Looking at the results of the precision studies in Table 2, the repeatability confirmed the high performance of the Ph. Eur. assay. Nevertheless, determination of the intermediate precision revealed the superiority of GAE% quantification via the FC assay, with a 1.3% relative standard deviation (RSD), compared to THCD quantification via the Ph. Eur. assay with a 4.1% RSD. The easier handling of the FC assay compared to the Ph. Eur. assay could be the reason for the better repeatability of the results on different days.

Both assays were used as reference analyses for the establishment of NIR spectroscopic methods.

### 2.2. Ultra-High Performance Liquid Chromatography

**Figure 1:**
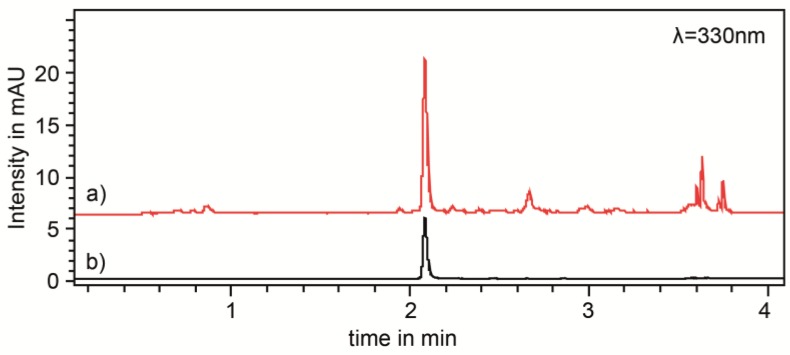
Chromatograms of (**a**) rosemary extract (red line) in 70% *v*/*v* ethanol (50 g/L) after 3 h continued stirred extraction, and (**b**) rosmarinic acid reference solution (black line), measured at 330 nm.

Nowadays, automatable methods like UHPLC-UV measurements are more common than wet chemical assays. This is because the sample preparation for UHPLC-UV measurement is often easier than for a wet chemical assay. Furthermore, fewer mistakes and variations in the analyses occur in UHPLC-UV. Also, in the present case, precision studies of the UHPLC-UV measurements of Rosmarini folium extracts obtained good repeatability (0.28% RSD) and excellent intermediate precision (0.55% RSD) for the determination of rosmarinic acid (RA) compared to the wet chemical assays (see Table 2). An example of a Rosmarini folium extract chromatogram compared to a RA reference solution, which was used for external calibration, is illustrated in Figure 1. Although the RA quantification showed such good results it is important to note that biological extracts are multi- substance mixtures of secondary metabolites. This is the reason that the Pearson correlations (Table 1) between the UHPLC-UV measurements and the wet chemical assays (0.955 and 0.953) were lower than the Pearson correlation between the wet chemical assays (0.966). Nevertheless, a high correlation between all three reference analysis methods was observed. Based on this fact, the reference analytical method of choice for the establishment of a NIR spectroscopic method should either be the UHPLC-UV analysis for the quantification of the single substance, RA, or the FC assay which was the better performing wet chemical assay (see Section 2.1) representing the plant extract as multi-substance mixture.

### 2.3. Near-Infrared Spectroscopy

**Figure 2:**
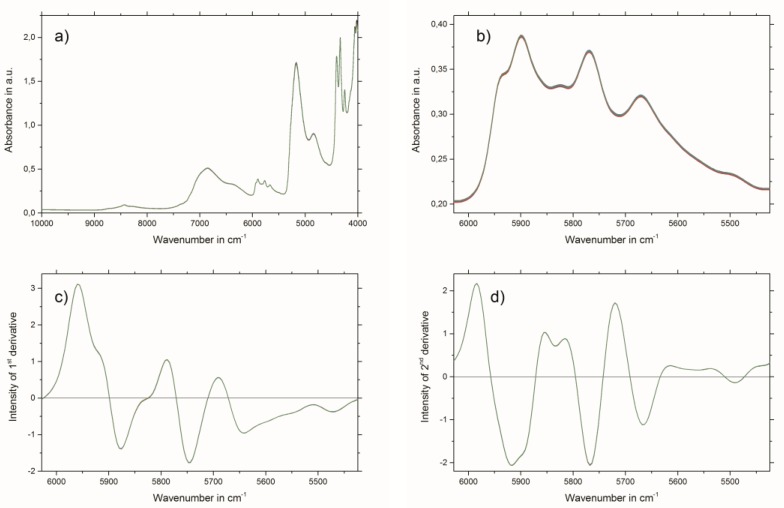
(**a**) Raw near-infrared (NIR) spectra of all 90 samples; (**b**) section from the raw NIR spectra showing the wavenumber region used for PLSR model calculation; (**c**) first derivate (13 smoothing points) and standard normal variate (SNV)-transformed NIR spectra region used for total hydroxycinnamic derivatives (THCD) in mg/kg and gallic acid equivalents (GAE)% PLSR model calculation; and (**d**) second derivate (23 smoothing points) and SNV-transformed NIR spectra region used for rosmarinic acid (RA)% PLSR model calculation.

Raw NIR spectra of all 90 samples are shown in Figure 2a. For the establishment of the PLSR models, uninformative and interfering spectral regions were excluded. Therefore, the best PLSR models were obtained by using the wavenumber region from 6028 to 5424 cm^−1^, which is illustrated in Figure 2b.

**Figure 3:**
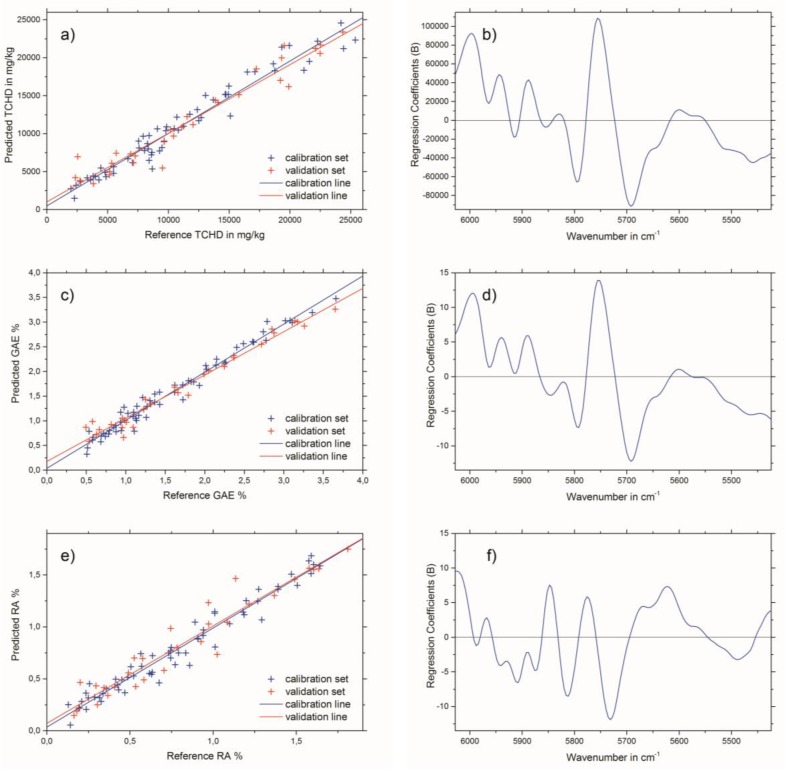
Predicted versus reference plots (left column) and regression coefficient plots (right column) for the best test-set validated PLSR models for: (**a**) and (**b**) THCD in mg/kg, (**c**) and (**d**) GAE%, and (**e**) and (**f**) RA%.

The results of the best PLSR models for THCD in mg/kg, GAE% and RA% are given in Table 3. The best spectral pretreatment for THCD in mg/kg and GAE% was the first derivative, using 13 smoothing points followed by applying standard normal variate (SNV) transformation to the selected wavenumber region (see Figure 2c). The best spectral pretreatment for the determination of RA% was the second derivative, with 23 smoothing points followed by applying SNV transformation to the selected wavenumber region (see Figure 2d). Predicted versus reference plots, and the regression coefficient plots for the three PLSR models are shown in Figure 3a,b for THCD in mg/kg, Figure 3c,d for GAE%, and Figure 3e,f for RA%.

**Figure 4:**
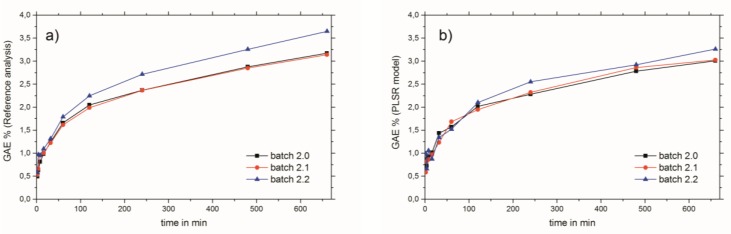
Monitoring of the extraction process of Rosmarini folium via (**a**) Folin–Ciocalteu reference analysis and (**b**) NIR spectroscopy.

NIR bands of the wavenumber region used (see Figure 2b) which have an influence on the PLSR model calculations can be considered mainly as aromatic and unsaturated 2νCH. This is due to the diverse, but nevertheless chemically similar, structures of the THCD, the total phenolic content, and the RA content. Therefore, other bands which emerge from overtones or combinations of OH, CC, and CO vibrations can be excluded for the establishment of the PLSR model [21] The criteria for the successful end of the extraction process of Rosmarini folium is to access the extraction plateau. This can be easily achieved by the reference analyses methods (see Figure 4a). However, these need analysis time and manpower, as well as chemicals, and are therefore not suited for real-time at-line monitoring. All three NIR spectroscopic PLSR models also showed satisfactory results for the monitoring of the extraction process of Rosmarini folium. Since the reference analyses were all correlated (see Table 1) the application of just one of the PSLR models was required to obtain the desired result. Therefore, it is suggested that the best PLSR model should be applied. The model for GAE% showed the best performance, as indicated by comparing the values for root mean square error of cross validation (RMSECV) or root mean square error of prediction (RMSEP) to the given calibration ranges in Table 3. These values were almost in the range of the lower edge of the calibration line for THCD in mg/kg and RA%. For GAE%, the RMSECV or RMSEP were much smaller than the minimum value of the calibration range. Therefore, it is suggested that the PLSR model for GAE% be applied for monitoring the extraction process of Rosmarini folium. Figure 4b illustrates the extraction monitoring using NIR spectroscopy for GAE% prediction via the PLSR model. In direct comparison, Figure 4a shows monitoring via the reference analysis method.

Although the reference method has better intermediate precision with 0.033 GAE%, the PLSR model with a RMSEP of 0.18 GAE% is absolutely satisfactory for monitoring the extraction process of Rosmarini folium. Furthermore, in contrast to the common off-line reference analyses, NIR spectroscopic process monitoring has convincing advantages since its operation is non-destructive, contact-free, pollution-free, does not require any additional solvents, saves energy and is highly cost-effective [15].

## 3. Materials and Methods

### 3.1. Chemicals

Ethanol (99.9%, LiChrosolv for liquid chromatography), acetonitrile (99.9%, LiChrosolv Reag. Ph. Eur., gradient grade for liquid chromatography) were purchased from Merck Millipore (Darmstadt, Germany). Hydrochloric acid (0.5 N), sodium nitrite (>98%), sodium molybdate dihydrate (>99.5%) and sodium hydroxide tablets (>98%) were bought from Carl Roth GmbH + Co. KG (Karlsruhe, Germany). Rosmarinic acid (>99%), Folin–Ciocalteu′s phenol reagent (2N), gallic acid (>97.5%), formic acid (98–100%, Suprapur for trace analysis) and sodium carbonate anhydrous (>99.8%) were obtained from Sigma Aldrich Handels GmbH (Vienna, Austria). H_2_O was purified using a Mili-Q^®^ reference water purification system from Merck Millipore. *Rosmarinus officinalis* L. leaves (Rosmarini folium) were collected in the wild at Lake Garda (Italy).

### 3.2. Extraction and Sampling

Dried *Rosmarinus officinalis* L. leaves were weighed (25 g ± 1 g) and extracted with 500 mL 70% (*v*/*v*) ethanol. The extraction was done in a 500 mL glass vessel with constant stirring using a color squid (IKA, Staufen im Breisgau, Germany). The extraction time lasted a maximum of 12 h. Three different sampling schedules were planned, and each was conducted three times (total: nine batches). For each sampling 1.5 mL were taken. The specimen-taking schedules are presented in Table 4. The numbering of the batches was done in following way: #(sampling). #(batch). Therefore, the three batches for each sampling schedule were denoted as 1.0, 1.1 and 1.2 or 2.0, 2.1 and 2.2 or 3.0, 3.1 and 3.2 for sampling 1, sampling 2, or sampling 3, respectively.

### 3.3. Wet Chemical Assays

#### 3.3.1. European Pharmacopoeia

The THCD of plant extracts were determined according to the procedure reported by the Ph. Eur. [6] with some modifications: 1.0 mL of sample solution was taken to which 2.0 mL of 0.5 M hydrochloric acid, 2 mL of nitrite–molybdate solution (10 g of sodium nitrite and 10 g of sodium molybdate in 100 mL water) and 2 mL of 1 M sodium hydroxide solution were added. The mixture was made up to 10 mL with water. Absorbance was measured with a Jenway Genova Plus Life Science Spectrophotometer (Cole-Parmer, Stone, United Kingdom) at 505 nm and quantification was performed with RA as an external standard calibration. Every extraction sample and calibration sample was prepared in the same way as described above. The repeatability and intermediate precision were determined according to ICH (international council for harmonization of technical requirements for pharmaceuticals for human use) guidelines [22,23]. Therefore, three samples with low, medium, and high THCD content were analyzed for five days, three times per day.

#### 3.3.2. Folin–Ciocalteu

The total phenolic content of plant extracts in GAE% was determined using FC reagent according to the procedure reported by Singleton, Orthofer, and Lamuela-Raventos [24] with some modifications. First, 1.5 mL of H_2_O was placed in a macrocuvette (PMMA, Brand, Germany). Next, 100 µL of sample solution, 100 µL of FC′s phenol reagent, and 1.3 mL of Na_2_CO_3_ were added. The mixture was heated to 60 °C for 30 min. After the heating procedure the samples were cooled for 20 min to room temperature. Absorbance was measured with the spectrophotometer at 750 nm. Quantification of the total phenolic content was performed by an external calibration with gallic acid. All extraction samples and calibration samples were prepared in the same way as described above. The repeatability and intermediate precision were determined according to ICH guidelines [22,23]. Therefore, three samples with low, medium, and high GAE% were analyzed for five days, three times per day.

### 3.4. Ultra-High Performance Liquid Chromatography

UHPLC analysis of RA was performed with an Agilent 1290 Infinity II LC Systems (Agilent Technologies, Santa Clara, CA, USA) equipped with a binary pump (G7120A), an autosampler (G7167B), a column oven (G7116B), and a DAD (diode array detector) (G7117A). Separation of RA was achieved by using an Agilent ZORBAX Eclipse Plus C18, Rapid Resolution HD 2.1 × 50 mm, 1.8 µm (Agilent Technologies, CA, USA) as the analytical column. The mobile phase was a composition of 0.5% formic acid in water (*v*/*v*, eluent A) and 0.5% formic acid in acetonitrile (*v*/*v*, eluent B). A gradient program was performed using the following steps (min/% eluent B): 0/15, 6/70, 6.1/100, 8/100, 8.1/15, and 10/15. The temperature of the column oven was set to 40 °C and detection of RA was performed at 330 nm. The flowrate was 1 mL/min and the injection volume was 1 µL. Quantification was performed using the external standard method. The repeatability and intermediate precision were determined according to ICH guidelines [22,23]. Therefore, three samples with low, medium and high RA% were analyzed for five days, three times per day.

### 3.5. Near-Infrared Spectroscopy

NIR spectra were measured using the NIRFlex N-500 FT-NIR spectrometer (Buchi, Flawil, Switzerland) with the NIRFLex Liquids cell and the cuvette cell add-on. The operating software was NIR Ware 1.4.3010 (Buchi, Flawil, Switzerland). Spectra of the extracts were recorded using precision cells (Hellma GmbH & Co. Kg., Müllheim, Germany) made of Quartz SUPRASIL 300 with a light path of 1 mm at a cell temperature of 35 °C. The spectral resolution was set to 8 cm^−1^ and the measurements were carried out in the wavenumber region from 10,000 to 4000 cm^−1^. Three replicates for each sample were recorded, with 32 scans each. Spectra were averaged to one representative spectrum per sample.

### 3.6. Multivariate Data Analysis

MVA was performed using The Unscrambler X Version: 10.5 software (CAMO Software, Oslo, Norway). First, transmittance spectra were transformed to absorbance spectra in order to establish PLSR models. The following spectral pretreatments were applied, alone or in combination, to identify the best PLSR model: baseline correction, SNV transformation [25], multiplicative scatter correction (MSC) [26], and first or second derivative. Savitzky-Golay derivatives [27] (quadratic polynomial) were optimized by variation of the smoothing points. Furthermore, spectral regions which contained no relevant information or even worsened the PLSR models were excluded. The NIPALS algorithm [26] was applied for calculating the PLSR models. For each of the three reference methods (Ph. Eur., FC, and UHPLC) an optimized model was established. The models were validated by full cross validation (CV), also known as leave one out cross validation (LOOCV) [28], and test-set validation (TSV). For TSV, batches 2.0, 2.1, and 2.2 (30 samples) were set as the independent test-set, and batches 1.0, 1.1, 1.2, 3.1, 3.2 and 3.3 (60 samples which included the extreme values) were used as the calibration set. The number of factors was chosen at the suggestion of the software, The Unscrambler X, and examined by expert reviewing. The calculated PLSR models were evaluated with the following parameters: root mean square error of calibration (RMSEC), RMSECV for CV, RMSEP for TSV, coefficient of determination (R^2^), regression coefficients, and the number of factors.

## 4. Conclusions

A fast analysis of the extraction process for the production of phytopharmaceuticals is indispensable in terms of economic viability and quality assurance. Common analytical methods which can be used for Rosmarini folium extraction monitoring are time- and resource-intensive and do not fulfill the requirement for real-time process control. However, the NIR spectroscopic analysis method provides a fast and non-invasive alternative analysis method, which can subsequently be implemented for on- or in-line process monitoring of the phytoextraction of Rosmarini folium.

## Figures and Tables

**Table 1:** Pearson correlations of the reference analyses.

	Ph. Eur.	FC	UHPLC
**Ph. Eur.**	1	-	-
**FC**	0.966	1	-
**UHPLC**	0.955	0.953	1

**Table 2:** Parameters of the precision studies of the reference analysis.

	Ph. Eur.	FC	UHPLC
**Repeatability in % RSD**	0.12	1.1	0.28
**Intermediate precision in % RSD**	4.1	1.3	0.55
**Repeatability** (absolute)	16 *	0.028 **	0.0017 ***
**Intermediate precision** (absolute)	593 *	0.033 **	0.0028 ***

* THCD mg/kg, ** GAE%, *** RA%

**Table 3:** Parameters of the established partial least squares regression PLSR models.

Reference Analysis	Ph. Eur.		FC		UHPLC	
**Samples**	90		90		90	
**Outliers**	0		0		0	
	CV	TSV	CV	TSV	CV	TSV
**R^2^_calibration_**	0.95	0.95	0.97	0.97	0.94	0.95
**R^2^_validation_**	0.94	0.94	0.96	0.96	0.94	0.93
**RMSEC ^(a)^**	1425 *	1308 *	0.14 **	0.13 **	0.11 ***	0.09 ***
**RMSECV ^(b)^ or RMSEP ^(c)^**	1527 *	1632 *	0.16 **	0.18 **	0.12 ***	0.13 ***
**Factor**	3	4	3	4	4	4
**Calibration range**	1975–25378 *		0.494–3.660 **		−1.810 ***	

* THCD mg/kg, ** GAE%, *** RA%, ^(a)^ root mean square error of calibration, ^(b)^ root mean square error of cross validation, ^(c)^ root mean square error of prediction.

**Table 4:** Sampling schedule for extraction experiments.

Sampling 1	Sampling 2	Sampling 3
1.5 min	2 min	2.5 min
3 min	4 min	5 min
6 min	8 min	10 min
12 min	16 min	20 min
24 min	32 min	40 min
45 min	60 min	50 min
90 min	120 min	80 min
180 min	240 min	150 min
360 min	480 min	300 min
720 min	660 min	600 min

## References

[1] Cragg G.M., Newman D.J. (2013). Natural products: A continuing source of novel drug leads. Biochim. Et Biophys. Acta (BBA) - Gen. Subj..

[2] Veeresham C. (2012). Natural products derived from plants as a source of drugs. J. Adv. Pharm. Technol. Res..

[3] Uhlenbrock L., Sixt M., Tegtmeier M., Schulz H., Hagels H., Ditz R., Strube J. (2018). Natural Products Extraction of the Future—Sustainable Manufacturing Solutions for Societal Needs. Processes.

[4] Kirchler C.G., Pezzei C.K., Beć K.B., Henn R., Ishigaki M., Ozaki Y., Huck C.W. (2017). Critical Evaluation of NIR and ATR-IR Spectroscopic Quantifications of Rosmarinic Acid in Rosmarini folium Supported by Quantum Chemical Calculations. Planta Med..

[5] European Medicines Agency Committee on Herbal Medicinal Products (HMPC) https://www.ema.europa.eu/en/committees/committee-herbal-medicinal-products-hmpc#.Accessed.

[6] EDQM Council of Europe (2016). European Pharmacopoeia.

[7] Ditz R., Gerard D., Hagels H., Igl N., Schäffler M., Schulz H., Stürtz M., Tegtmeier M., Treutwein J., Chemat F. (2017). Phytoextracts: Proposal towards a new comprehensive Research Focus.

[8] Sixt M., Gudi G., Schulz H., Strube J. (2018). In-line Raman spectroscopy and advanced process control for the extraction of anethole and fenchone from fennel (Foeniculum vulgare L. MILL.). Comptes Rendus Chim..

[9] Gavan A., Colobatiu L., Mocan A., Toiu A., Tomuta I. (2018). Development of a NIR Method for the In-Line Quantification of the Total Polyphenolic Content: A Study Applied on Ajuga genevensis L. Dry Extract Obtained in a Fluid Bed Process. Molecules.

[10] Wang P., Zhang H., Yang H., Nie L., Zang H. (2015). Rapid determination of major bioactive isoflavonoid compounds during the extraction process of kudzu (Pueraria lobata) by near-infrared transmission spectroscopy. Spectrochim. Acta Part A Mol. Biomol. Spectrosc..

[11] Wu Z., Sui C., Xu B., Ai L., Ma Q., Shi X., Qiao Y. (2013). Multivariate detection limits of on-line NIR model for extraction process of chlorogenic acid from Lonicera japonica. J. Pharm. Biomed. Anal..

[12] Wu Y., Jin Y., Ding H., Luan L., Chen Y., Liu X. (2011). In-line monitoring of extraction process of scutellarein from Erigeron breviscapus (vant.) Hand-Mazz based on qualitative and quantitative uses of near-infrared spectroscopy. Spectrochim. Acta Part A Mol. Biomol. Spectrosc..

[13] Chen X., Li Y., Chen Y., Wang L., Sun C., Liu X. (2009). Study on fast quality control in extracting process of Paeonia lactiflora using near infrared spectroscopy. China J. Chin. Mater. Med..

[14] Hu T., Li T., Nie L., Zang L., Zang H., Zeng Y. (2017). Rapid monitoring the water extraction process of Radix Paeoniae Alba using near infrared spectroscopy. J. Innov. Opt. Health Sci..

[15] Siesler H.W., Ozaki Y., Kawata S., Heise H.M. (2002). Near-infrared spectroscopy: Principles, Instruments, Applications.

[16] Aeschbach R., Löliger J., Scott B.C., Mucia A., Butler J., Halliwell B., Aruoma O.I. (1994). Antioxidant actions of thymol, carvacrol, 6-gingerol, zingerone and hydroxytyrosol. Food Chem. Toxicol..

[17] Schönbichler S.A., Falser G.F.J., Hussain S., Bittner L.K., Abel G., Popp M., Bonn G.K., Huck C.W. (2014). Comparison of NIR and ATR-IR spectroscopy for determination of the capaxity of Primulae flox cum calycibus. Anal. Methods.

[18] Rover M.R., Brown R.C. (2013). Quantification of total phenols in bio-oil using the Folin–Ciocalteu method. J. Anal. Appl. Pyrolysis.

[19] Colowick S.P., Kaplan N.O., Abelson J.N., Simon M.I., Packer L. (1999). Methods in Enzymology.

[20] Waterhouse A.L. (2002). Determination of Total Phenolics. Curr. Protoc. Food Anal. Chem..

[21] Kirchler C.G., Pezzei C.K., Bec K.B., Mayr S., Ishigaki M., Ozaki Y., Huck C.W. (2017). Critical evaluation of spectral information of benchtop vs. portable near-infrared spectrometers: Quantum chemistry and two-dimensional correlation spectroscopy for a better understanding of PLS regression models of the rosmarinic acid content in Rosmarini folium. Analyst.

[22] Guidance for Industry: Q2B Validation of Analytical Procedures: Methodology. https://www.fda.gov/downloads/Drugs/GuidanceComplianceRegulatoryInformation/Guidances/UCM073384.pdf.

[23] Shabir G.A. (2004). A practical approach to validation of HPLC methods under current good manufacturing practices. J. Valid. Technol..

[24] Singleton V.L., Orthofer R., Lamuela-Raventós R.M. (1999). [14] Analysis of total phenols and other oxidation substrates and antioxidants by means of folin-ciocalteu reagent. Methods Enzym..

[25] Barnes R.J., Dhanoa M.S., Lister S.J. (1989). Standard Normal Variate Transformation and De-trending of Near-Infrared Diffuse Reflectance Spectra. Appl. Spectrosc..

[26] Geladi P., MacDougall D., Martens H. (1985). Linearization and Scatter-Correction for Near-Infrared Reflectance Spectra of Meat. Appl. Spectrosc..

[27] Savitzky A., Golay M.J.E. (1964). Smoothing and Differentiation of Data by Simplified Least Squares Procedures. Anal. Chem..

[28] Haaland D.M., Thomas E.V. (1988). Partial least-squares methods for spectral analyses. 1. Relation to other quantitative calibration methods and the extraction of qualitative information. Anal. Chem..

